# Supplementary material for: Content-rich biological network constructed by mining PubMed abstracts
Source: BMC Bioinformatics. 2004 Oct 8;5:147. doi: 10.1186/1471-2105-5-147 (PMC528731; doi:10.1186/1471-2105-5-147)
Supplement: Additional File 5 — The original Chilibot query results of the term "long-term potentiation (LTP)" and 22 other terms, limiting the latest references analyzed to the years 1990, 1995, 2000, and 2004. [file 1471-2105-5-147-S5.bz2 › chilibotAdditionalFile5/ltp2004/html/ARC_SYNAPTOTAGMIN.html]

 


 **ARC** and **SYNAPTOTAGMIN** 
  
Found 4 abstracts in PubMed,  **4 abstracts were retrieved and analyzed**.  


---

 Search Google  |
 PDF files only 
|  EDU domain only 

---

**Interactive relationship** (e.g. stimulation, inhibition, etc)

**Parallel relationship** (e.g. studied together, co-existance, homology, etc.)

- On the other hand, neuromolecular studies showed increased phosphorylation of neuromodulin, increased expression of tissue type plasminogen activator,  **synaptotagmin**  IV and  **arc** , which are assumed to play roles in neural outgrowth and synaptogenesis.  Ref: 10464775 Nihon Shinkei Seishin Yakurigaku Zasshi, 1999
